# Niosomal Encapsulation of Phenolic-Rich Extract from Fermented Corn Bran: Optimization, Characterization, and In Vitro Evaluation

**DOI:** 10.3390/foods15173071

**Published:** 2026-08-30

**Authors:** Mehdi Akbari, Clara Gomez-Urios, Seyed Hadi Razavi, María J. Esteve, Jesús Blesa

**Affiliations:** 1 Bioprocess Engineering Laboratory (BPEL), Department of Food Science, Engineering and Technology, College of Agriculture and Natural Resources, University of Tehran, Karaj 31587-77871, Iran; akbari.mehdi@ut.ac.ir; 2 Nutrition and Food Science, Faculty of Pharmacy and Food Science, University of Valencia, Avenida Vicent Andrés Estellés, 22, 46100 Burjassot, Spain; clara.urios@uv.es (C.G.-U.); jesus.blesa@uv.es (J.B.)

**Keywords:** niosomes, phenolic compounds, encapsulation efficiency, controlled release, food nanotechnology

## Abstract

This study developed and optimized a niosomal delivery system for fermented corn bran extract (FCBE) to enhance its stability and evaluate its antioxidant potential for nutritional and health applications. Niosomes were prepared using a central composite design (CCD) to assess the effects of surfactant hydrophilic-lipophilic balance (HLB) and cholesterol content on encapsulation efficiency (EE) and particle size. The optimized formulation, obtained at an HLB of 13.5 and 92 mg cholesterol, achieved an EE of approximately 56% and a mean particle size of 205 nm. Antioxidant activity assays showed that the DPPH scavenging capacity of encapsulated FCBE was comparable to that of the free extract, whereas ABTS scavenging activity was lower for the encapsulated extract (9.54%) than for the free extract (16.8%). This lower apparent ABTS activity was attributed to the restricted release of phenolic compounds from the niosomal vesicles in the aqueous assay medium, rather than to a loss of their intrinsic antioxidant capacity. In vitro release studies confirmed that niosomal encapsulation provided a more controlled and sustained release profile. Stability evaluation demonstrated good preservation under refrigerated conditions, while higher temperatures and acidic pH reduced encapsulation efficiency over time. These findings highlight the potential of niosomes as carriers of plant-derived bioactive compounds for functional food and pharmaceutical applications.

## 1. Introduction

Corn bran, the outer layer of the corn kernel, is a rich source of bioactive compounds, particularly phenolic acids, which have been shown to exhibit various health-promoting properties, including antioxidant, anti-inflammatory, anti-diabetic, and anti-cancer effects [1]. However, these bioactive compounds are often sensitive to environmental factors such as heat, light, and oxygen during processing, which can result in their degradation and reduced bioavailability. Exposure to these stressors may induce alterations in the chemical structure and composition of the bioactive compounds, thereby diminishing their therapeutic properties [2,3].

In the human body, these compounds are subject to additional challenges that may further affect their stability and absorption. Following ingestion, bioactive compounds are exposed to varying pH levels and digestive enzymes throughout the gastrointestinal tract. The acidic conditions of the stomach and the alkaline environment of the small intestine can induce the degradation or chemical alteration of some compounds. Furthermore, digestive enzymes can metabolize certain bioactive compounds, leading to structural changes that may diminish their biological activity [4,5].

To address these challenges, different encapsulation techniques have been investigated to protect sensitive bioactive compounds and improve their stability and bioavailability. Among the various encapsulation technologies developed to protect sensitive bioactive compounds, lipid-based nanocarriers have attracted considerable attention because of their ability to improve the stability, bioavailability, and controlled release of antioxidants [6]. Liposomes have been widely investigated for the encapsulation of phenolic compounds owing to their excellent biocompatibility and capacity to protect bioactive compounds from oxidation and degradation during processing and gastrointestinal digestion. However, their practical application may be limited by phospholipid oxidation, relatively high production costs, and lower physicochemical stability during storage [7,8]. In contrast, niosomes, which are vesicular systems composed of non-ionic surfactants, offer several advantages, including greater chemical stability, lower production costs, ease of large-scale production, and the ability to encapsulate both hydrophilic and lipophilic compounds. These characteristics make niosomes particularly attractive carriers for natural antioxidants and phenolic-rich extracts intended for functional food and nutraceutical applications. In addition, niosomes can protect encapsulated antioxidants against environmental stressors, improve their stability during storage and gastrointestinal transit, and provide sustained release, thereby enhancing their potential biological efficacy [6,9,10].

Several studies have investigated the encapsulation of plant-derived bioactive compounds using niosomes. For example, bioactive compounds extracted from rice bran [11], fermented soybean [12], and roasted coffee residues [13] have been successfully encapsulated using niosomal systems. More recently, Tween/Span-based niosomes have been investigated for the encapsulation of phenolic compounds. Zolghadri et al. (2023) developed gallic acid-loaded niosomes using different Tween 60/Span 60 ratios in combination with cholesterol, demonstrating their potential for the encapsulation and controlled release of this phenolic compound [14]. Similarly, Span 60/Tween 60/cholesterol niosomes have recently been investigated as carriers for curcumin, further supporting the applicability of these surfactant-based vesicular systems for the delivery of polyphenolic compounds [15]. However, to the best of our knowledge, the encapsulation of bioactive compounds extracted from fermented corn bran using niosomes has not yet been explored.

In our previous study [16], we demonstrated that fermentation of corn bran enhanced its total phenolic content and antioxidant activity. The fermentation process can promote the release of bound phenolic compounds from the plant matrix, resulting in an extract enriched with bioactive compounds. However, phenolic compounds are susceptible to degradation during processing, storage, and gastrointestinal transit, which may limit their functional applications. Therefore, an appropriate delivery system is needed to protect these bioactive compounds, improve their stability, and potentially enhance their bioavailability. Building on these findings, the present study aims to develop and optimize a niosomal delivery system for a phenolic-rich extract obtained from fermented corn bran using response surface methodology, and to comprehensively evaluate its physicochemical characteristics, encapsulation efficiency, antioxidant activity, in vitro release behavior, and storage stability. We hypothesized that encapsulation of fermented corn bran extract (FCBE) within niosomes would enhance the stability and provide a controlled release of the bioactive compounds, thereby potentially improving their health-promoting effects.

## 2. Materials and Methods

### 2.1. Materials

Sorbitan monostearate (Span 60), polyethylene glycol sorbitan monolaurate (Tween 20), cholesterol, chloroform, ethanol, 2,2-diphenyl-1-picrylhydrazyl (DPPH), and 2,2′-azinobis 3-ethylbenzothiazoline-6-sulfonic acid (ABTS) were purchased from Sigma-Aldrich (Steinheim, Germany).

### 2.2. Preparation of Fermented Corn Bran Extract

The FCBE used in this study was prepared through solid-state fermentation. Briefly, corn bran was inoculated with Lactobacillus reuteri at an initial cell density of 1 × 10^7^ CFU/mL. The fermentation process was carried out at 37 °C for 72 h. Following fermentation, the corn bran was subjected to aqueous extraction to recover the extract. The resulting extract was then centrifuged, and the supernatant was collected, freeze-dried, and stored at −20 °C until further analysis and experimental use.

### 2.3. Central Composite Design (CCD)

Response Surface Methodology (RSM) was employed to optimize niosomal formulations using a central composite design (CCD). Two independent variables, including hydrophilic-lipophilic balance (HLB) value (X_1_) and cholesterol content (X_2_), were investigated at five levels. Thirteen experimental runs were conducted, including six replicates at the center point. The resulting niosomal suspensions were evaluated for encapsulation efficiency (EE, Y_1_) and vesicle size (Y_2_). All measurements were performed in triplicate, and the mean values were used as the response variables (Table 1).

### 2.4. Niosome Preparation

Niosomes were prepared using the thin film hydration technique, based on the method reported by Gunes et al. (2017) [17] with minor modifications. Surfactant (10 mM Span 60, Tween 20, or a mixture of Span 60 and Tween 20) and cholesterol were dissolved in 10 mL of chloroform and transferred to a 250 mL round-bottom flask. The total surfactant concentration was fixed at 10 mM in all formulations, and the cholesterol levels of 25, 50, 75, 100, and 125 mg corresponded to 39.27, 56.39, 65.98, 72.12, and 76.38 mol%, respectively, relative to the total molar amount of surfactant(s) and cholesterol. Subsequently, 10 mL of FCBE was added to the flask. The solvent was then evaporated using a rotary evaporator (Laborota 4003, Heidolph Instruments GmbH & Co., Schwabach, Germany) under reduced pressure at 50 °C with agitation at 80 rpm, resulting in the formation of a thin lipid film on the inner wall of the flask. The thin film was hydrated with 20 mL of distilled water at 50 °C under gentle rotation at 20 rpm for 30 min until a white niosomal suspension was obtained. The suspension was subsequently sonicated for 20 min in an ultrasonic bath to reduce vesicle size. Finally, the niosomal formulations were transferred to vials and stored at refrigeration temperature until further evaluation.

### 2.5. Determination of Encapsulation Efficiency (EE)

One mL of niosomal suspension was centrifuged (Centrifuge 5810 R, Eppendorf, Germany) at 27,000 rpm for 90 min. The supernatant was collected, and the concentration of phenolic compounds in the supernatant was quantified by measuring absorbance at 280 nm using a PerkinElmer, Waltham, MA, USA). The selection of 280 nm was based on the characteristic UV absorption of phenolic compounds, particularly ferulic acid, the major bioactive phenolic compound in fermented corn bran extract. Concentrations were determined based on a previously established standard calibration curve. Encapsulation efficiency (EE) was subsequently calculated using the following equation:

$$EE\;\left(\%\right)=\left[\frac{\left(C_{0}-C_{S}\right)}{C_{0}}\right]\;\times 100$$

In the above equation, C_S_ is the concentration of phenolic compounds present in the supernatant after centrifugation, and C_0_ is the initial concentration of phenolic compounds [18].

### 2.6. Vesicle Size Determination

The hydrodynamic diameter of niosomes encapsulating FCBE was measured by dynamic light scattering (DLS) analysis using a particle size analyzer at 25 °C. The samples were diluted 24 h after preparation by adding 10 mL of distilled water to 50 µL of the niosomal suspension. The diluted samples were then transferred to the instrument’s cuvette and analyzed to determine the mean vesicle size. The Polydispersity Index (PdI) was used as a measure of the size distribution of the niosomal formulation. A PdI value below 0.3 was considered indicative of a relatively homogeneous and narrowly distributed colloidal system [10].

### 2.7. Fourier-Transform Infrared (FT-IR) Spectroscopy

The FT-IR spectra of the phenolic extract, surfactant, cholesterol, and the prepared niosomes were obtained separately and compared using a spectrometer (Thermo Nicolet, Madison, WI, USA). The samples were scanned in transmission mode over the wavenumber range of 650 to 4000 cm^−1^ with a resolution of 4 cm^−1^.

### 2.8. Morphological Studies

The morphology of niosomes was examined using transmission electron microscopy (TEM). For sample preparation, a drop of niosomal suspension was placed onto a carbon-coated copper grid and allowed to adhere for 1 min. Excess suspension was carefully removed with filter paper. Negative staining was then performed by applying 2% phosphotungstic acid for one minute, followed by removal of the excess stain with filter paper. After air drying, the samples were imaged using TEM, according to the procedure described by Pando et al. (2013) [19].

### 2.9. Antioxidant Activity

#### 2.9.1. Radical Scavenging by DPPH Assay

The DPPH free radical scavenging capacity of FCBE and EFCBE (Encapsulated fermented corn bran extract) was evaluated according to the method reported by Anticona et al. (2021) [20] with modifications. A 50 µL aliquot of the FCBE or niosomal suspension was combined with 1450 µL of a DPPH solution (60 µmol/L) in glass test tubes. The mixture was vigorously shaken and incubated for 30 min in the dark at room temperature. Then, the absorbance of the mixture was measured at 515 nm. The procedure was repeated in triplicate, and the scavenging activity percentage of the samples was calculated using the equation below:

DPPH^•^ scavenging activity (%) = [(A_517_ blank − A_517_ Extract)/A_517_ blank] × 100

#### 2.9.2. Radical Scavenging by ABTS Assay

A stock solution of ABTS^•+^ was prepared by mixing 25 mL of ABTS solution (7 mmol/L) with 440 µL of potassium persulfate solution (140 mmol/L), followed by dilution with distilled water until an absorbance of 0.7 at 734 nm was achieved after equilibration at 30 °C. For the assay, 30 µL of the FCBE or EFCBE was added to 3 mL of the ABTS^•+^ solution and incubated at 30 °C for 10 min. Then, the absorbance value was measured at 734 nm. Distilled water (30 µL) was used as a blank. ABTS^•+^ scavenging activity (%) was calculated using the following equation:

ABTS^•+^ scavenging activity (%) = [(A_734_ Blank − A_734_ Extract)/A_734_ Blank] × 100

### 2.10. In Vitro Release

The release profile of EFCBE from niosomes was assayed using the dialysis technique in phosphate-buffered solution. Briefly, 10 mL of niosomal solution was put into a cellulose acetate dialysis bag and immersed in 100 mL of phosphate buffer at 37 °C and pH 7.4 to provide a controlled aqueous environment representative of the intestinal pH for 48 h. This condition was selected to evaluate the in vitro release behavior of FCBE under intestinal-like conditions rather than to fully simulate the gastrointestinal environment. At predetermined time intervals (2, 4, 6, 8, 10, 12, 18, 24, and 48 h), one milliliter of the immersing solution was withdrawn and immediately replaced with an equal volume of fresh buffer equilibrated at the same temperature and pH. The collected samples were then analyzed using a spectrophotometer at 280 nm, and the release of the EFCBE from the niosomes was plotted using the standard curve [21].

### 2.11. Stability Studies

Niosomal suspensions were filled into glass vials and stored at three different temperatures (4, 25, and 45 °C) for two months to assess thermal stability. The EE was assessed at predetermined intervals throughout the storage period. Additionally, the stability of the niosomes was evaluated across various pH levels (3, 5, and 7). For the pH stability study, the niosomal suspensions were adjusted to initial pH values of 3, 5, and 7 using phosphoric acid. The samples were stored at 4 °C for 1 month, and their encapsulation efficiency (EE) was evaluated at 0, 15, and 30 days to assess niosome stability under different initial pH conditions [22].

### 2.12. Statistical Analysis

Data were statistically analyzed using the SPSS Inc. software, version 32.0.0. (IBM, Chicago, IL, USA). Statistical significance was calculated using one-way analysis of variance (ANOVA), followed by Duncan’s multiple range test for post hoc comparisons. Differences were considered significant at *p* < 0.05.

## 3. Results and Discussion

### 3.1. RSM Approach for Optimizing the Niosomal Formulations

HLB value is a crucial factor influencing the formation and physicochemical properties of niosomes. In this study, five HLB values ranging from 4.5 to 16.5 were selected. Span 60 (HLB 4.5) and Tween 20 (HLB 16.5) were used individually to achieve these extreme HLB values. Intermediate HLB values were attained by preparing blends of Tween 20 and Span 60 in appropriate ratios. Another factor considered in response surface design was the amount of cholesterol. Cholesterol can influence the behavior and structure of vesicles due to its interaction with non-ionic surfactants. Various concentrations of cholesterol, up to 125 mg, have been used to prepare niosomal suspensions.

The experimental data are presented in Table 2. The results indicate that the formulation corresponding to the second treatment, with an HLB value of 13.5 and a cholesterol content of 100 mg, exhibited the highest EE of 57.49%. In contrast, the lowest EE (13%) was observed in the sixth formulation, which contained 25 mg of cholesterol and an HLB value of 10.5. Regarding particle size, the largest vesicles (365 nm) were obtained in the fourth treatment, whereas the smallest vesicles (108 nm) were observed in the tenth treatment.

#### 3.1.1. The Effect of Variables on the Niosome Particle Size

The ANOVA results (Table S1) indicated that the linear model developed by the software provided the best fit for the data, with an F-value of 7.92 and a *p*-value of 0.008. The quality of the predicted linear model was also examined through the coefficient of determination R^2^, based on the estimated coefficients. The final equation describing the relationship between the variables and niosome particle size was derived as follows:

$$Particle\;size=237.69-47.89\;X_{1}+22.53\;X_{2}$$

**Figure 1:**
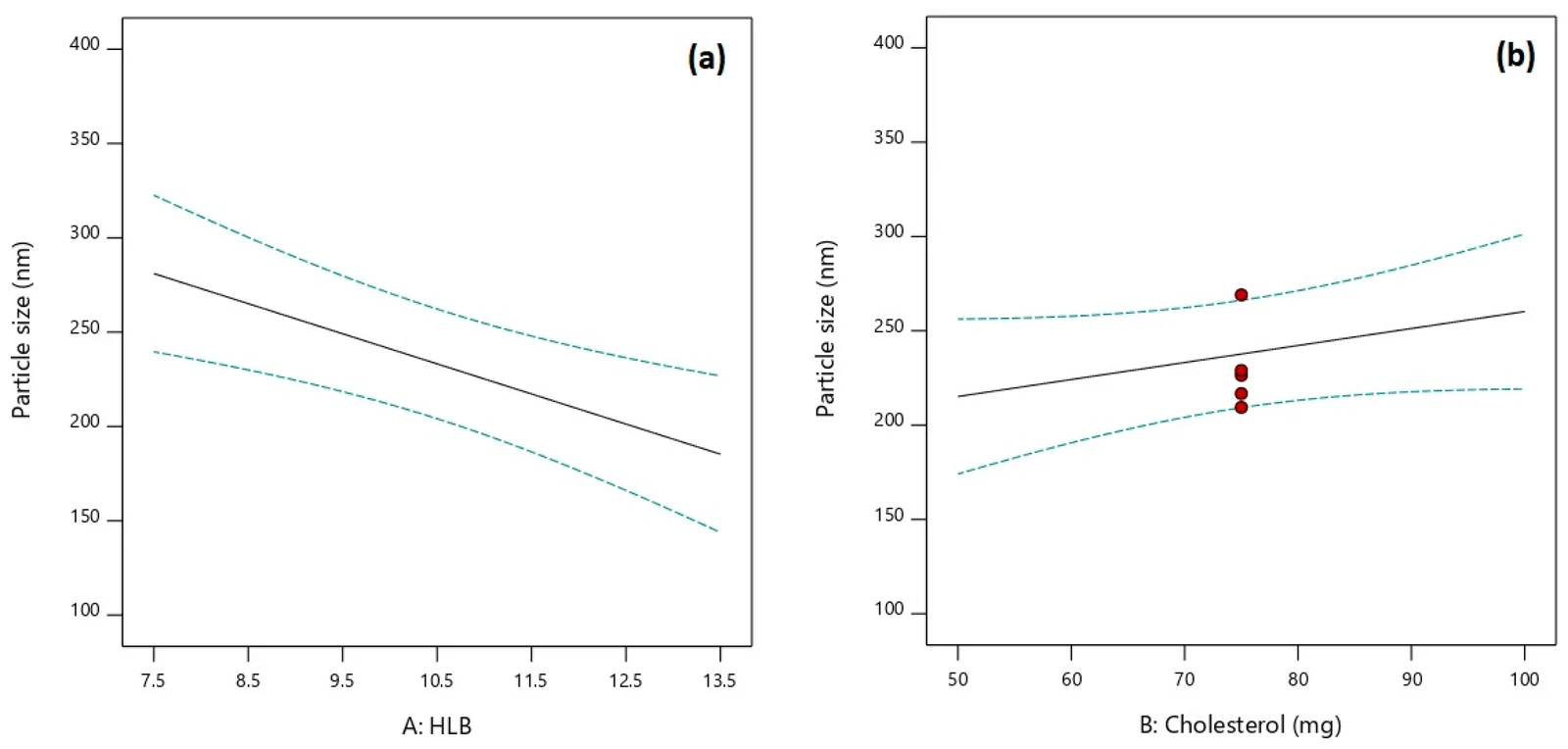
The effect of (**a**) HLB value and (**b**) cholesterol content on the particle size of niosomes.

This model equation describes the influence of the independent variables, the HLB value (X_1_) and the amount of cholesterol (X_2_), on the particle size of niosomes prepared with different formulations. The negative coefficient of X_1_ indicated that higher HLB values resulted in smaller niosome particle sizes (Figure 1a). Surfactants with higher HLB values possess larger polar head groups, which may promote a molecular geometry favorable for the formation of vesicular structures. In addition, possible interactions between the functional groups of the bioactive compounds and the polar head groups of the surfactants may contribute to niosomal cohesion and affect vesicle size [18].

On the other hand, the positive coefficient of X_2_ suggested that increasing the cholesterol content in the niosomal formulation could lead to an increase in the particle size of the niosomes in suspension (Figure 1b). However, this result is not consistent with those obtained by Nowroozi et al. (2018) [23], who reported that higher cholesterol levels reduced particle size in niosomes containing Span 60 and Brij 72. The authors attributed this reduction to enhanced hydrophobicity of niosome layers induced by cholesterol, which lowered the surface free energy and consequently resulted in smaller vesicles.

The interaction between cholesterol and nonionic surfactants is not limited to changes in bilayer hydrophobicity; cholesterol can also modify surfactant packing, bilayer organization, and the phase behavior of the membrane. In particular, cholesterol can interact with the hydrophobic regions of surfactant molecules and alter the ordering and organization of their hydrocarbon chains, thereby affecting bilayer stability, fluidity, and permeability [24,25,26]. The extent and direction of these effects depend on the physicochemical characteristics of the surfactant, including its HLB, hydrophobic chain structure, and phase transition behavior. For example, molecular dynamics simulations of Span 60/cholesterol bilayers demonstrated that increasing cholesterol concentration substantially altered bilayer structure and stability, including changes in molecular area, bilayer thickness, chain conformation, and hydrogen bond interactions [25]. Therefore, it can be concluded that the effect of cholesterol on niosome particle size is dependent on its specific interactions with the surfactants employed, which can modify the molecular packing and self-assembly of the bilayer and consequently influence vesicle formation and growth. This surfactant-dependent behavior may explain the different effects of cholesterol on particle size observed among different niosomal systems, highlighting the critical role of surfactant type in modulating the physical characteristics of niosomes.

#### 3.1.2. The Effect of Variables on the EE

For the response of EE, the ANOVA (Table S2) showed an F-value of 19.32 and a *p*-value less than 0.0006, indicating that the predicted model was highly significant. The quadratic model was identified as the most appropriate fit for this response. The coefficient of determination (R^2^) and adjusted-R^2^ were 0.93 and 0.88, respectively, demonstrating a strong correlation between the model and the experimental data. The resulting equation describing EE is shown in Equation (1).

(1) $$EE\left(\%\right)=+50.66+4.93\;X_{1}+10.83\;X_{2}+1.88\;X_{1}X_{2}-5.66\;X_{1}^{2}-5.23\;X_{2}^{2}$$

The positive coefficient of the interaction term X_1_X_2_ in the equation showed a synergistic effect between HLB and cholesterol content on EE of niosomes. This is consistent with previous studies showing that cholesterol enhances EE by stabilizing the niosomal membrane and interacting with the hydrophilic heads of surfactants, especially those with higher HLB values. For instance, Agarwal et al. (2004) [27] reported that EE increased with increasing cholesterol content and varied according to surfactant HLB, highlighting the combined influence of both factors on vesicle properties. Similarly, recent findings in nanomedicine formulations demonstrated that niosomes prepared with higher cholesterol ratios and surfactants of higher HLB exhibited significantly improved EE, confirming the synergistic effect of these two variables on vesicular drug loading [28].

**Figure 2:**
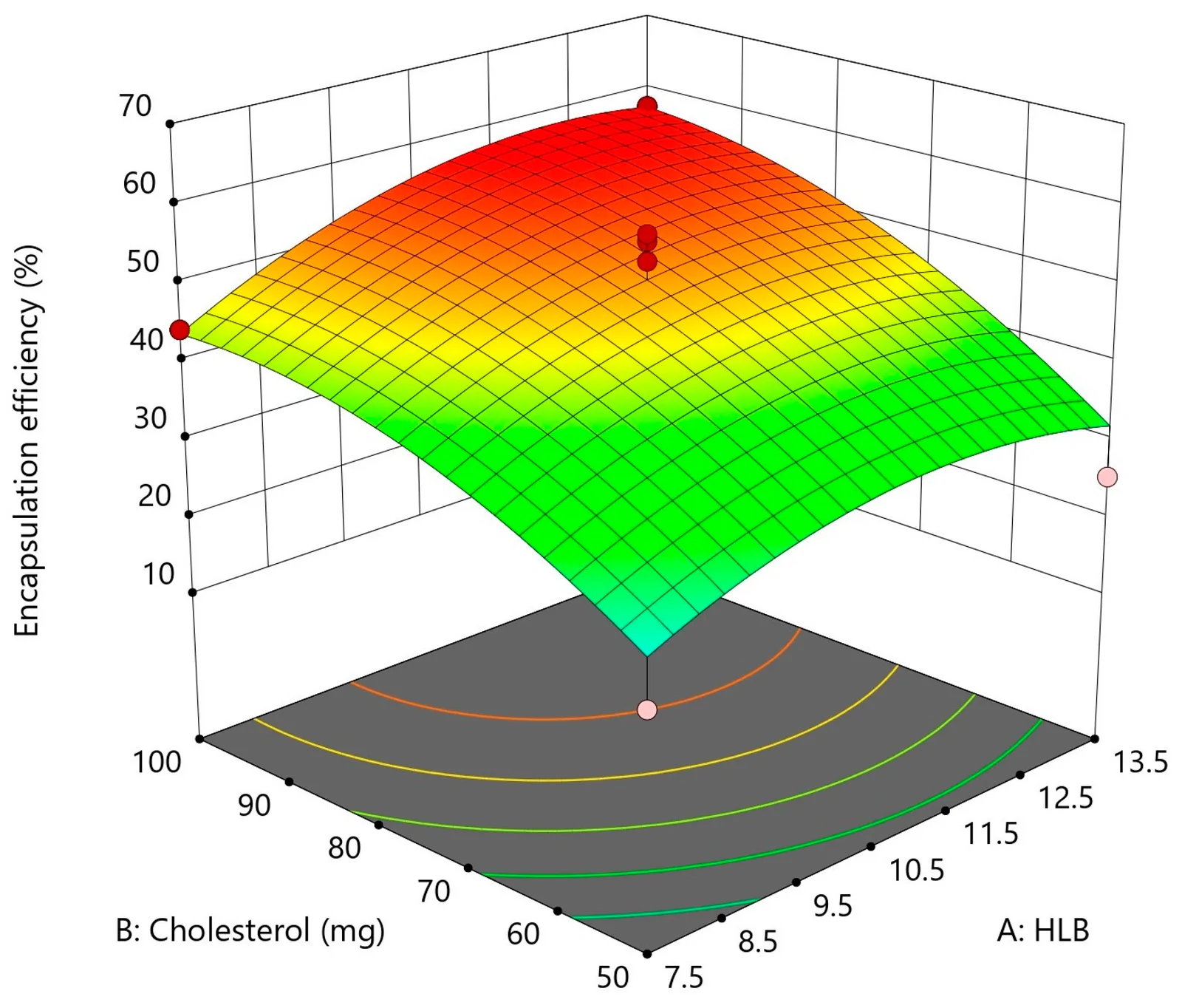
The combined effect of HLB value and cholesterol amount on the encapsulation efficiency and particle size of niosomes.

Niosomes prepared using polysorbate surfactants, which possess high HLB values, are capable of effectively entrapping both hydrophilic and hydrophobic bioactive compounds. The polar head groups (hydrophilic regions) of polysorbate surfactants facilitate interactions with phenolic and lactonic compounds, while their non-polar fatty acid chains interact with hydrophobic molecules. However, as shown in a three-dimensional response surface plot (Figure 2), an increase in the surfactant’s HLB value beyond a certain threshold adversely affects the EE of phenolic compounds. This decline occurs due to the fact that surfactants with very high HLB values have a larger hydrophilic part compared to their hydrocarbon chains, making it difficult to form stable niosomes. Excessive hydration caused by highly hydrophilic surfactants can destabilize the niosome structure. The critical packing parameter (CPP) and the balance between the hydrophilic and hydrophobic domains within the surfactant molecule are essential determinants of niosome formation and stability [23].

#### 3.1.3. Optimized Formulation

According to Table 3, the optimal conditions for producing niosomal suspensions with the highest EE and the smallest particle size were determined to be an HLB value of 13.5 nm and a cholesterol content of 92 mg (approximately 70.5 mol%). Under these conditions, the model predicted an EE of 56% and a particle size of 205 nm. Finally, the optimized niosomal formulation was prepared, and its EE and particle size were experimentally measured. The observed values were in close agreement with the predicted results, confirming the validity of the optimization. Therefore, the optimized formulation was selected for further investigations.

### 3.2. FT-IR

**Figure 3:**
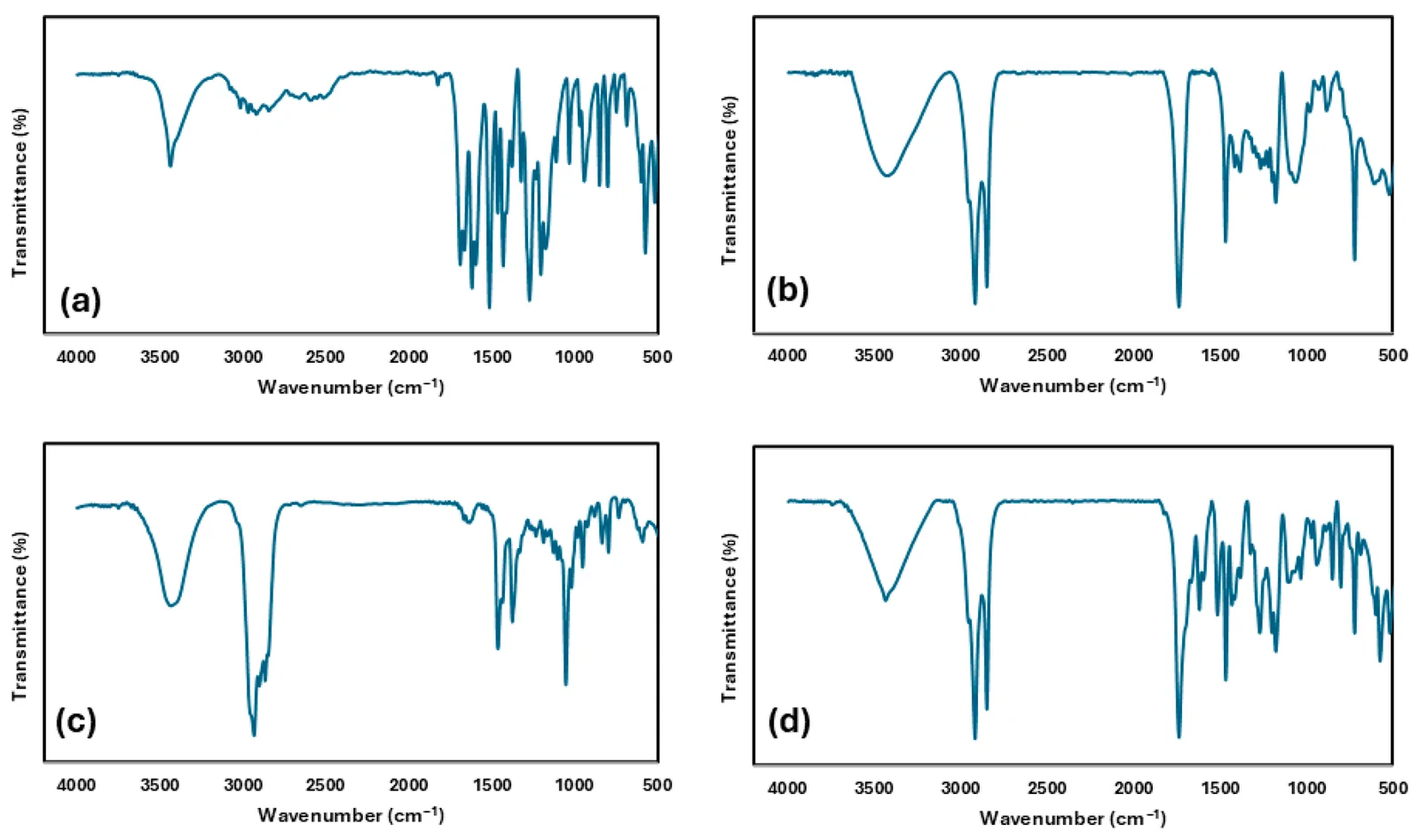
FT-IR spectra of (**a**) phenolic extract, (**b**) Span 60/Tween 20 surfactant mixture, (**c**) cholesterol, and (**d**) optimized niosomes.

The chemical bonds and functional groups of the FCBE, cholesterol, the Span 60/Tween 20 surfactant mixture used in the optimized formulation, and the FCBE encapsulated within the niosomes (optimized formulation) are shown in Figure 3. FCBE showed absorbance peaks at 3437 cm^−1^ (OH stretching), 1620–1432 cm^−1^ (C=C stretching), and 1274, 1205, and 1176 cm^−1^ (CO stretching). These peaks were also observed in the FT-IR spectrum of the EFCBE. Similarly, the main characteristic peaks of cholesterol and the surfactants were also present in the spectrum of the optimized niosomes. No clear shift in the peaks or appearance of new peaks was observed, particularly in the O–H stretching region. This suggests that the encapsulation process did not cause major chemical changes in FCBE or the other formulation components. However, weak interactions such as hydrogen bonding between the bioactive compounds and the polar groups of the surfactants cannot be completely ruled out based on FT-IR results alone, as the broad O–H band may hide small changes.

Cholesterol showed distinctive peaks at 3433 cm^−1^ (OH stretching), 2933–2867 cm^−1^ (CH asymmetric and symmetric stretching), 1465 cm^−1^ (CH bending), and 1056 cm^−1^ (CO stretching). Span 60/Tween 20 surfactant mixture used in the optimized formulation displayed absorption bands at 3425 cm^−1^ (O–H stretching), 2918 cm^−1^ (CH_2_ asymmetric stretching), 2850 cm^−1^ (CH_2_ symmetric stretching), and 1738 cm^−1^ (C=C stretching).

### 3.3. Transmission Electron Microscopy (TEM)

**Figure 4:**
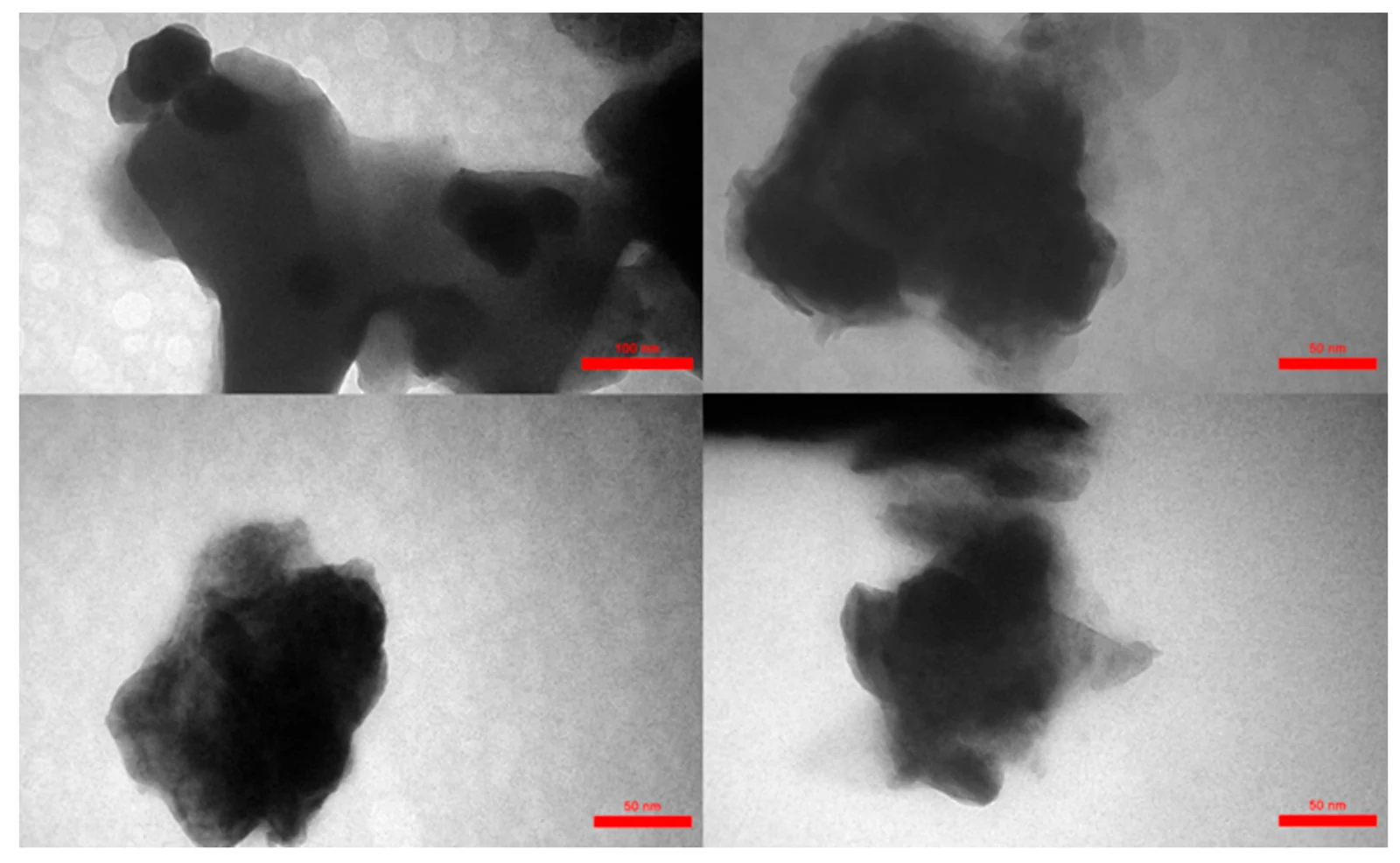
Transmission Electron micrographs of optimized niosomal suspension.

A TEM image of the optimized niosomal suspension is depicted in Figure 4, providing a detailed visualization of the formulated niosomes. The images revealed vesicular structures with an average size of approximately 200 nm, consistent with the particle size determined by DLS (205 nm). The optimized formulation also exhibited a relatively low PDI of 0.25, indicating a relatively narrow particle-size distribution and limited size heterogeneity. The close agreement between the two characterization techniques supports the consistency of the particle size characterization. However, the difference in their measurement principles should be considered: DLS determines the hydrodynamic diameter of particles dispersed in liquid, whereas TEM provides direct visualization of vesicles after sample preparation [29].

The nanoscale dimensions of the vesicles are critical for their functional performance in drug delivery systems, as particles of this size may exhibit enhanced cellular uptake and improved permeation through biological membranes. This size range may also support controlled and sustained release of encapsulated compounds, which could potentially enhance therapeutic outcomes and reduce potential systemic side effects. Additionally, the increased surface area provided by nanosized vesicles may facilitate greater interaction with target cells and tissues, potentially improving bioavailability and therapeutic efficacy.

### 3.4. Antioxidant Activity

**Figure 5:**
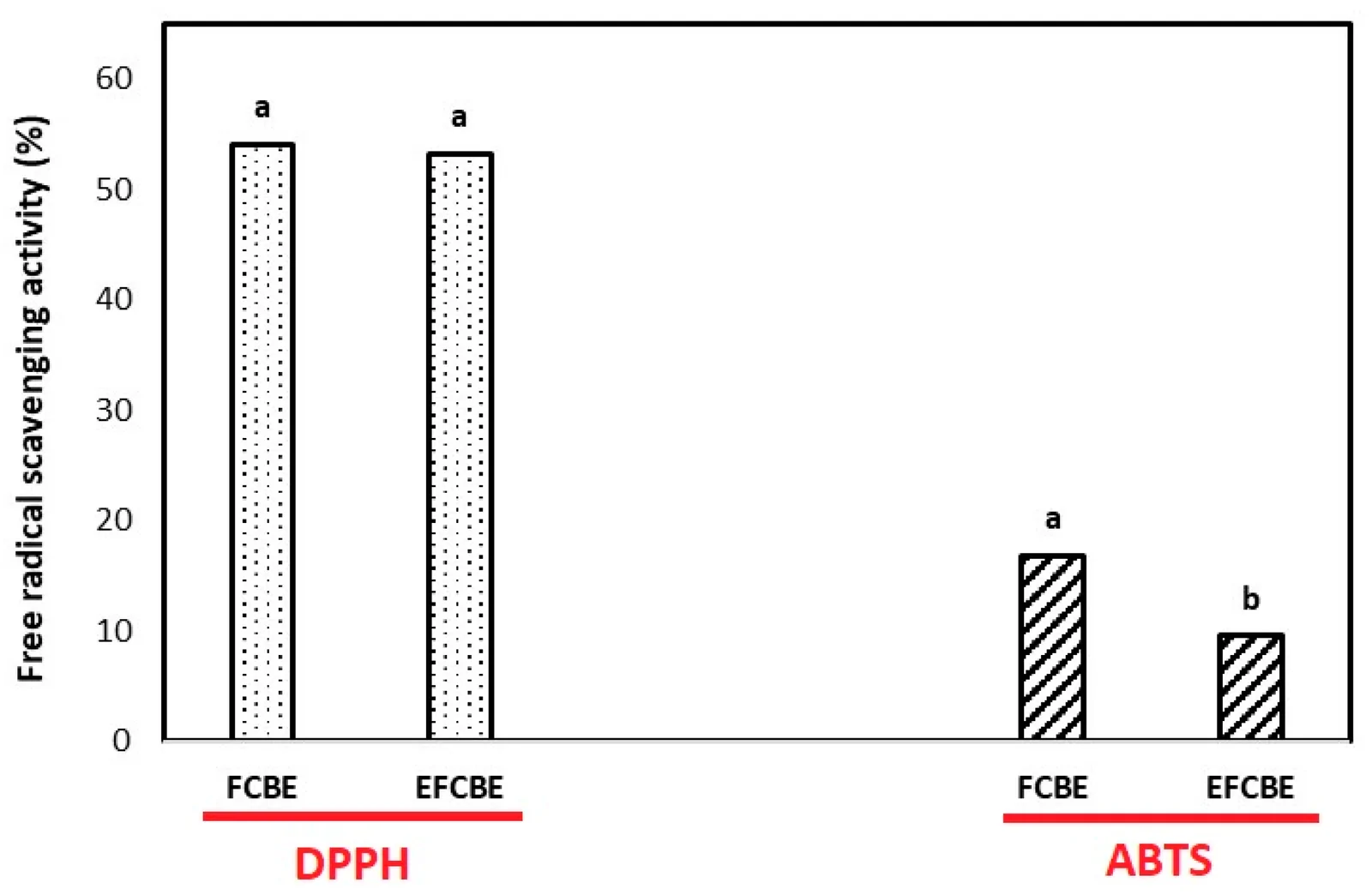
Radical scavenging capacity of fermented corn bran extract (FCBE) and encapsulated fermented corn bran extract (EFCBE) determined by DPPH and ABTS assays. Different lowercase letters indicate significant differences between the samples (*p* < 0.05).

The antioxidant activity of FCBE and EFCBE was evaluated using the DPPH and ABTS assays (Figure 5). The results obtained from the DPPH assay demonstrated that the DPPH radical scavenging activity of the EFCBE was similar to that of the free FCBE at the same concentration. This result indicated that encapsulation within niosomes does not alter the antioxidant activity of the phenolic compounds present in the extract. Upon exposure to organic solvents such as ethanol, the structural integrity of the niosomes is disrupted, leading to the release of phenolic compounds, including phenolic acids, which subsequently exert their antioxidant effects. Similar observations were reported by Machado et al. (2021) [6], who found that the antioxidant activity of resveratrol encapsulated in Tween 80:Span 80 niosomes was not significantly different from that of free resveratrol in solution.

On the other hand, according to the results obtained from the ABTS assay, it was observed that the antioxidant activity of the phenolic extract encapsulated in niosomes was lower than that of the control solution. This could be due to the protective effect of the niosome membrane on the phenolic compounds. Since the ABTS evaluation is performed in an aqueous environment, the vesicles remain stable, and the phenolic compounds cannot be released and fully exhibit their antioxidant properties. These findings are consistent with the results reported by Feng et al. (2017) [30], who found that the ABTS radical scavenging activity of catechin encapsulated in niosomes was lower than that of the control sample.

### 3.5. In Vitro Release

**Figure 6:**
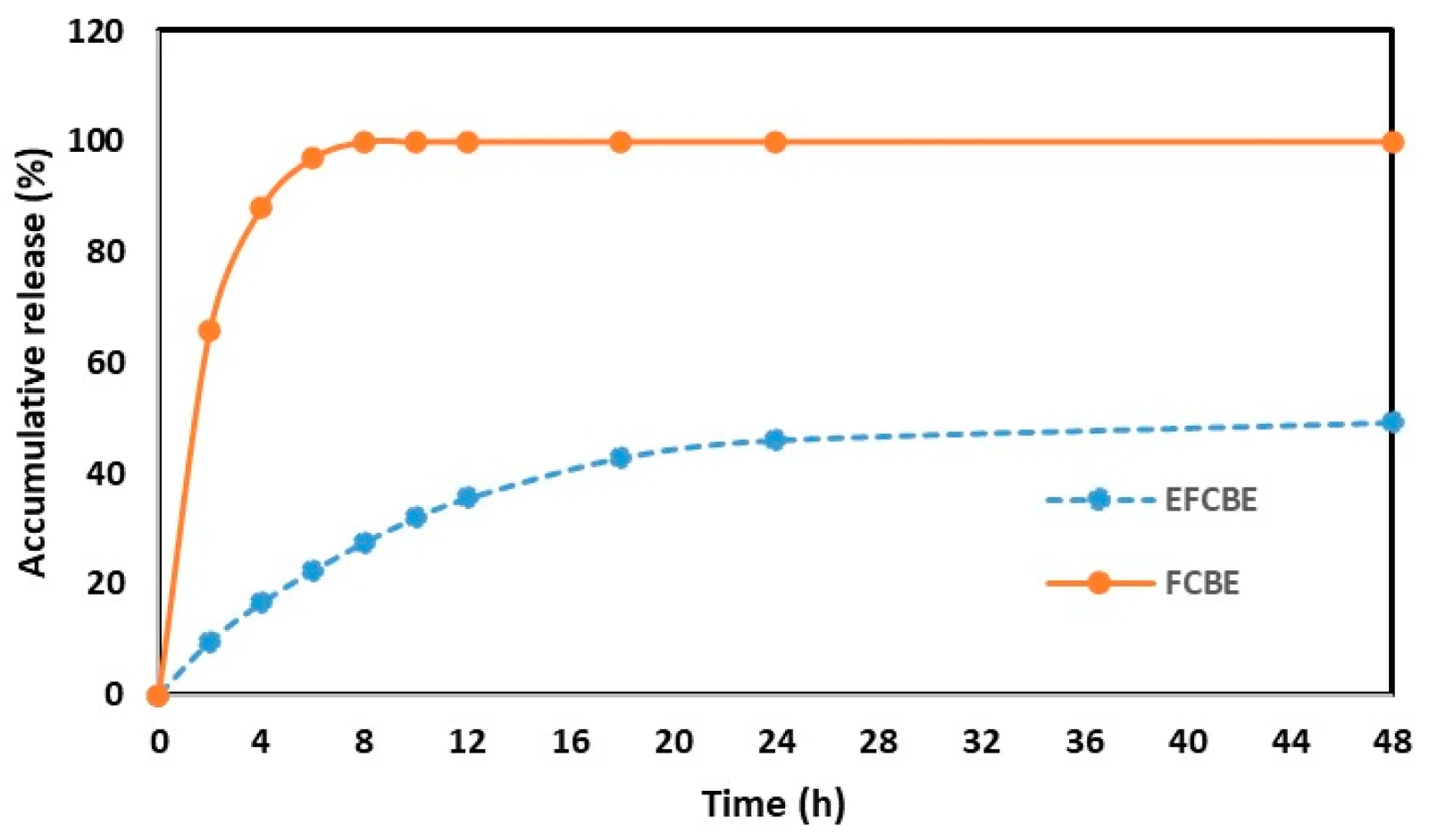
Release pattern of fermented corn bran extract in its free form (FCBE) and encapsulated within niosomes (EFCBE) over 48 h.

The results of the release test for the FCBE and EFCBE are illustrated in Figure 6. The percentage of phenolic extract released in the free form reached approximately 100% over six hours, whereas the amount released from the encapsulated phenolic extract during this period was around 22%. This indicated the protective effect of the niosome membrane on the bioactive compounds present in the phenolic extract. Additionally, the release of the phenolic extract encapsulated in niosomes occurred continuously and without explosive release. The fastest leakage of bioactive compounds from the extract loaded in niosomes was observed during the initial release phase, specifically within the first two hours, where approximately 10% of the release occurred. These results clearly demonstrated that encapsulating the phenolic extract in niosomes resulted in a more stable release of the bioactive compounds present in the extract. According to the obtained results, the highest release rate of the encapsulated extract was 50% over a period of 48 h.

### 3.6. Stability Studies

**Figure 7:**
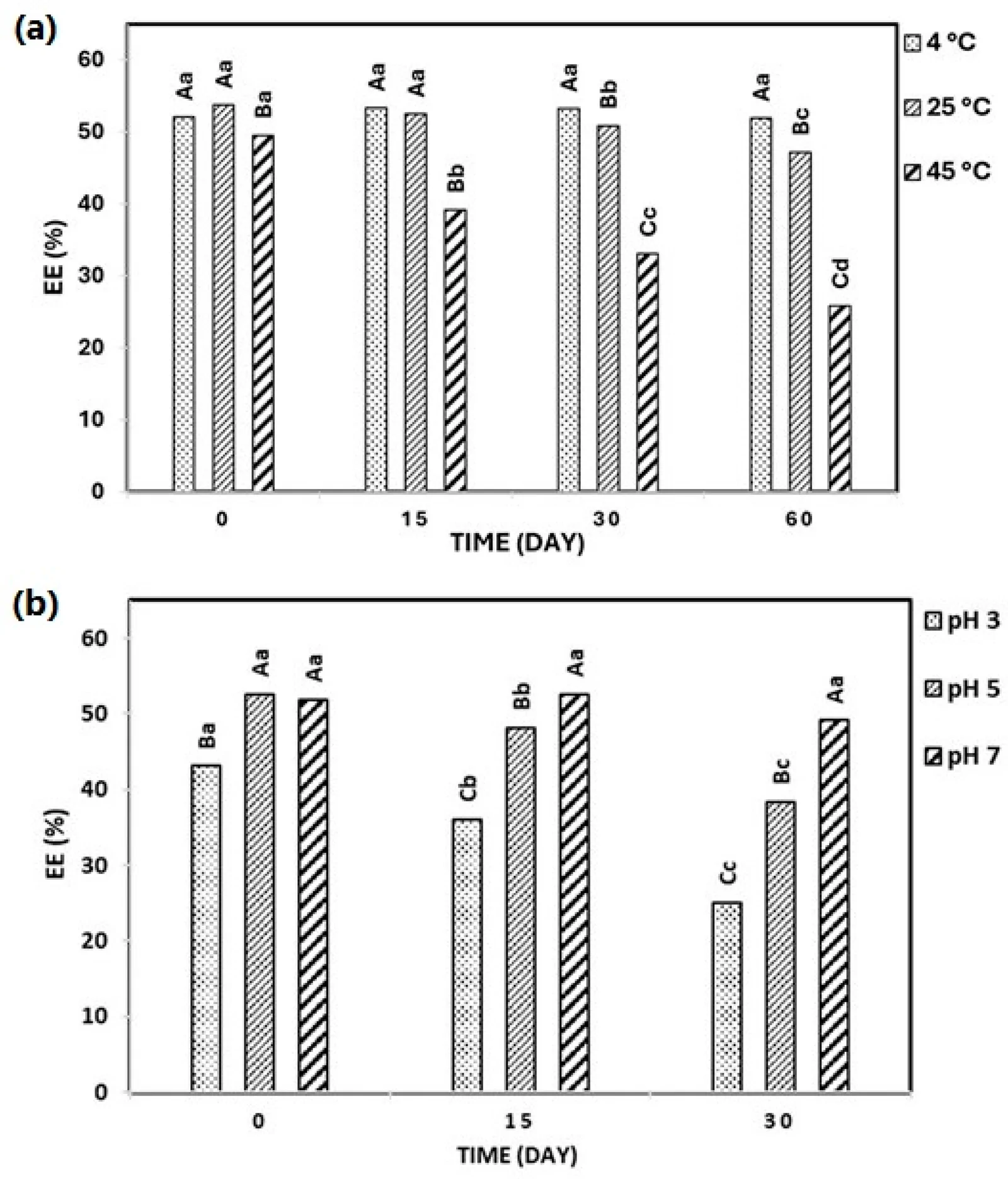
Stability of encapsulated fmented corn bran extract (EFCBE) at (**a**) different temperatures (4, 25, and 45 °C) and (**b**) different pH levels (3, 5, and 7). The stability of EFCBE at different pH values (3, 5, and 7) during one month of storage is shown in Figure 7b. The lowest EE was consistently observed at pH 3, indicating reduced stability of the niosomal system under acidic conditions. This decrease may be associated with changes in surfactant organization and interactions between the encapsulated phenolic compounds and the niosomal membrane, which can affect membrane integrity and compound retention [31,32]. Overall, these results indicate that both storage temperature and pH influence the stability of EFCBE, with refrigerated storage and less acidic conditions providing greater retention of the encapsulated compounds. Different lowercase letters indicate significant differences during storage time at the same condi-tion (temperature or pH), while different uppercase letters indicate significant differences among conditions (temperatures or pH levels) at the same time (*p* < 0.05).

The stability of EFCBE at different storage temperatures (4, 25, and 45 °C) is shown in Figure 7a. No significant changes in EE were observed at 4 °C during storage, whereas EE decreased significantly after 30 days at 25 °C and after 15 days at 45 °C.

## 4. Conclusions

This study demonstrated the potential of niosomal encapsulation as an effective strategy to enhance the functionality and applicability of a phenolic-rich extract derived from fermented corn bran, an agro-industrial by-product with limited direct use in food systems. By focusing on formulation optimization, this work clarified the role of surfactant HLB value and cholesterol content in controlling encapsulation efficiency and vesicle size, providing practical guidance for the design of niosomal carriers suitable for complex plant-based extracts.

The results showed that niosomal encapsulation preserved the intrinsic antioxidant capacity of the phenolic-rich extract while modulating its release behavior. The lower ABTS scavenging activity observed for the encapsulated extract should therefore be attributed to the restricted release of phenolic compounds from the niosomal vesicles in the aqueous assay medium, rather than to a loss of antioxidant capacity. The sustained release profile observed for the encapsulated extract highlights the ability of niosomes to overcome common limitations associated with the direct application of free phenolic compounds, such as rapid release and reduced stability. In addition, stability studies indicated that the optimized formulation maintains acceptable performance under refrigerated storage, although higher temperatures and acidic pH conditions negatively affect encapsulation efficiency.

While the fermentation process was not part of the present study, the use of a phenolic-rich extract obtained from fermented corn bran, followed by its stabilization through niosomal encapsulation, demonstrates how existing bioactive-rich by-products can be further valorized into functional ingredients with improved stability and controlled release properties. This approach supports more efficient use of agricultural resources and contributes to waste reduction through value-added product development.

These findings also highlight the practical potential of the developed niosomal system as a delivery platform for phenolic-rich extracts. Previous studies have also reported advantages of niosomes, including high encapsulation capacity, controlled release, physicochemical stability, and relatively low production cost compared with conventional liposomal systems. These characteristics support their potential application in functional foods and nutraceutical formulation.

## Figures and Tables

**Table 1:** Independent and dependent variables of the central composite design.

Independent Variables	Symbol	Levels				
		−α	−1	0	1	+α
HLB value	X_1_	4.5	7.5	10	13.5	16.5
Cholesterol (mg *)	X_2_	25	50	75	100	125
**Responses**		**Units**		**Goals**		
Encapsulation efficiency	Y_1_	%		Maximize		
Vesicle size	Y_2_	nm		Minimize		

* The cholesterol amounts shown in mg correspond to 39.27, 56.39, 65.98, 72.12, and 76.38 mol%, respectively, calculated relative to the total molar amount of surfactant(s) and cholesterol at a fixed total surfactant concentration of 10 mM.

**Table 2:** Experimental design for the independent variables and response values.

Run	Independent Factors		Responces	
	X_1_: HLB	X_2_: Cholesterol (mg)	EE (%)	Particle Size (nm)
1	10.5	125	49.55	282.12
2	13.5	100	57.49	206.64
3	10.5	75	55.47	216.72
4	7.5	50	19.67	365.04
5	10.5	75	48.12	209.4
6	10.5	25	13.01	176.712
7	16.5	75	39.65	187.8
8	4.5	75	19.5	286.92
9	10.5	75	46.51	269.04
10	13.5	50	25.32	108.12
11	7.5	100	44.33	326.1
12	10.5	75	56.37	226.44
13	10.5	75	52.97	228.96

**Table 3:** Predicted and observed values for the optimized niosomal formulation.

Factor	Optimized Level		
HLB value	13.5		
Cholesterol (mg; mol%)	92; 70.5		
**Response**	**Expected**	**Observed**	**Residual**
Encapsulation efficiency (%)	56	51	5
Vesicle size (nm)	205	217	−12

## Data Availability

The original contributions presented in this study are included in the article/Supplementary Material. Further inquiries can be directed to the corresponding authors.

## Supplementary Materials

The following supporting information can be downloaded at: https://www.mdpi.com/article/10.3390/foods15173071/s1, Table S1. Analysis of variance for response surface linear model for particle size in niosomal suspensions. Table S2. Analysis of variance for response surface quadratic model for TPC.

## Abbreviations

The following abbreviations are used in this manuscript:

ABTS	2,2′-Azino-bis(3-ethylbenzothiazoline-6-sulfonic acid)
CCD	Central composite design
CFU	Colony-forming units
DLS	Dynamic light scattering
DPPH	2,2-Diphenyl-1-picrylhydrazyl
EE	Encapsulation efficiency
EFCBE	Encapsulated fermented corn bran extract
FCBE	Fermented corn bran extract
FT-IR	Fourier-transform infrared spectroscopy
HLB	Hydrophilic–lipophilic balance
RSM	Response surface methodology
TEM	Transmission electron microscopy
