# Supplementary material for: Niosomal Encapsulation of Phenolic-Rich Extract from Fermented Corn Bran: Optimization, Characterization, and In Vitro Evaluation
Source: Foods. 2026 Aug 30;15(17):3071. doi: 10.3390/foods15173071 (PMC13564954; doi:10.3390/foods15173071)
Supplement: Supplementary file 1 [file foods-15-03071-s001.zip › foods-4459222-supplementary.pdf]

Niosomal Encapsulation of Phenolic-Rich Extract from Fermented Corn Bran: Optimization, Characterization, and In Vitro Evaluation

Mehdi Akbari [1], Clara Gomez-Urios [2], Seyed Hadi Razavi [1,*], María J. Esteve [2,*] and Jesús Blesa [2]

[1] Bioprocess Engineering Laboratory (BPEL), Department of Food Science, Engineering and Technology, College of Agriculture and Natural Resources, University of Tehran, Karaj 31587-77871, Iran; akbari.mehdi@ut.ac.ir
[2] Nutrition and Food Science, Faculty of Pharmacy and Food Science, University of Valencia, Avenida Vicent Andrés Estellés, 22, 46100 Burjassot, Spain; clara.urios@uv.es (C.G.-U.); jesus.blesa@uv.es (J.B.)
* Correspondence: srazavi@ut.ac.ir (S.H.R.); maria.jose.esteve@uv.es (M.J.E.)

Supplementary Material

Table S1. Analysis of variance for response surface linear model for particles size in niosomal suspensions.

Source	Sum of Squares	df	Mean Square	F-value	p-value	Significance
Model	33608.51	2	16804.26	7.92	0.0087	significant
X_1- HLB	27515.68	1	27515.68	12.97	0.0048	
X_2- Cholesterol	6092.83	1	6092.83	2.87	0.1210	
Residual	21208.94	10	2120.89			
Lack of Fit	19070.40	6	3178.40	5.95	0.0531	not significant
Pure Error	2138.53	4	534.63			
Cor Total	54817.45	12				

Table S2. Analysis of variance for response surface quadratic model for TPC.

Source	Sum of Squares	df	Mean Square	F-value	p-value	Significance
Model	2771.57	5	554.31	19.32	0.0006	significant
X_1- HLB	291.17	1	291.17	10.15	0.0154	
X_2- Cholesterol	1406.38	1	1406.38	49.01	0.0002	
X_1X_2	14.10	1	14.10	0.4913	0.5060	
X_1^2	732.77	1	732.77	25.53	0.0015	
X_2^2	626.47	1	626.47	21.83	0.0023	
Residual	200.88	7	28.70			
Lack of Fit	123.67	3	41.22	2.14	0.2385	not significant
Pure Error	77.21	4	19.30			
Cor Total	2972.45	12				
